# Animal Models and “Omics” Technologies for Identification of Novel Biomarkers and Drug Targets to Prevent Heart Failure

**DOI:** 10.1155/2015/212910

**Published:** 2015-07-07

**Authors:** Yunlong Hou, Juan M. Adrian-Segarra, Manfred Richter, Natalia Kubin, Jaeyoung Shin, Isabella Werner, Thomas Walther, Markus Schönburg, Jochen Pöling, Henning Warnecke, Thomas Braun, Sawa Kostin, Thomas Kubin

**Affiliations:** ^1^Department of Cardiac Development and Remodelling, Max Planck Institute for Heart and Lung Research, Ludwigstrasse 43, 61231 Bad Nauheim, Germany; ^2^Department of Cardiac Surgery, Kerckhoff Clinic, Benekestrasse 2-8, 61231 Bad Nauheim, Germany; ^3^Department of Thoracic and Cardiac Surgery, Goethe University Frankfurt am Main, 60323 Frankfurt am Main, Germany; ^4^Department of Cardiac Surgery, Schüchtermann-Clinic, Ulmenallee 11, 49214 Bad Rothenfelde, Germany; ^5^University of Witten/Herdecke, Alfred-Herrhausen-Straße 50, 58448 Witten, Germany

## Abstract

It is now accepted that heart failure (HF) is a complex multifunctional disease rather than simply a hemodynamic dysfunction. Despite its complexity, stressed cardiomyocytes often follow conserved patterns of structural remodelling in order to adapt, survive, and regenerate. When cardiac adaptations cannot cope with mechanical, ischemic, and metabolic loads efficiently or become chronically activated, as, for example, after infection, then the ongoing structural remodelling and dedifferentiation often lead to compromised pump function and patient death. It is, therefore, of major importance to understand key events in the progression from a compensatory left ventricular (LV) systolic dysfunction to a decompensatory LV systolic dysfunction and HF. To achieve this, various animal models in combination with an “omics” toolbox can be used. These approaches will ultimately lead to the identification of an arsenal of biomarkers and therapeutic targets which have the potential to shape the medicine of the future.

## 1. Introduction

The need of the body for supply of nutrition and oxygen requires continuous cardiac contraction. Physiological workload such as endurance training and pregnancy increases ventricular mass in order to perpetuate the “status quo.” Ventricular architecture and the differentiation status of the myocardium are essentially maintained and are features of physiological hypertrophy [1–3]. When the heart is not able to respond physiologically, for example, due to coronary artery disease, arterial hypertension, or a cardiomyopathy, myocardial changes take place that affect the protein composition and protein localization on the cellular as well as on the extracellular level [4, 5]. This ventricular remodeling leads to the activation of an evolutionary conserved “fetal gene program.” Reactivation of this program is a potential strategy when the heart is challenged by unfavorable mechanical and metabolic workloads in order to prevent transition from compensated hypertrophy to HF [3, 6, 7]. However, this initially “programmed cell survival” may also lead to pathophysiological alterations and compromised pump function when hypertrophic signals are chronically released [3, 6, 8, 9]. It is quite clear that the understanding of this transition is of clinical importance in order to halt and hopefully revert adverse cardiac remodeling. However, the classical view of HF as a simple hemodynamic disorder puts emphasis on a strategy to reduce unfavorable workload and does not address the complex interplay of structure and function [10].

It has been previously demonstrated that a structure-function relationship exists in human patients with end stage HF [6, 8, 11–17]. The degree of myocardial dysfunction is inversely related to cardiomyocyte degeneration, fibrosis, and macrophage infiltration and suggests a mutual influence of cardiac structure and function. Importantly, the degree of postoperative recovery of patients with a poor* structure-function *relationship is less favourable [8, 9]. A consensus paper on cardiac remodeling stressed that the determination of the ejection fraction reliably initiates the treatment of HF but does not evoke consensus among physicians in the treatment regimen [18]. As a consequence, therapeutic interventions which only aim to improve cardiac output and blood flow do not necessarily target the altered ultrastructure of the heart and in addition do not necessarily prevent cardiac remodeling or attenuate HF. Therefore, this consensus paper concludes that “clinicians should understand the relationship between remodeling and HF progression” [18]. In addition, the list of genes and proteins known to be involved in HF is far from being complete and represents probably only the “tip of the iceberg” [15]. In order to achieve a more comprehensive understanding of the transition from compensated adaptation to HF the combination of state-of-the-art profiling techniques with adequate animal models should lead to the identification of disease relevant molecules [19, 20]. Here, we introduce animal models with critical features of human cardiac disease and relevant strategies to identify novel drug targets and biomarker utilizing proteome as well as transcriptome approaches.

## 2. Animal Models of Heart Failure Mimic the Human Cardiac Pathology

**Figure 1:**
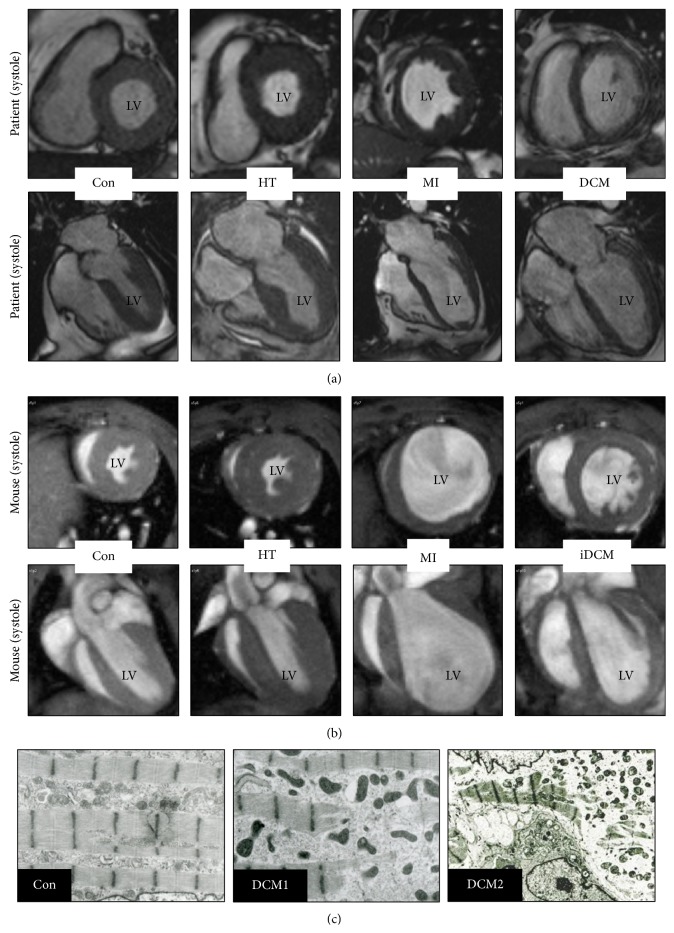
End-systolic midventricular short axis and long axis MRI frames of healthy and failing hearts from humans and mice. (a) MRI images from a healthy control (Con) and patients with aortic stenosis (HT), myocardial infarction (MI), and idiopathic dilated cardiomyopathy (DCM). (b) MRI images from mice 4 weeks after transaortic constriction (HT), 3 weeks after LAD ligation (LAD), and 6-month-old mice with a cardiac restricted overexpression of MCP-1 (iDCM). A healthy control animal is also shown (Con). Note the increase in size and ventricular mass of HT hearts while increases in heart size after MI and after development of (i) DCM are associated with ventricular thinning. Explanations are given in the text. (c) Electron microscopy pictures show different degrees of sarcomeric degeneration in patients with dilated cardiomyopathy (Con versus DCM1 and DCM2; EF < 30%). Human MRI images and electron micrographs were kindly provided by Professor Georg Bachmann and by Dr. Viktoria Polyakova, respectively. Mouse MRI images are adapted and modified from the “Venia legendi” work of J. Pöling.

Under physiological workload all cardiac cavities are maintained in a “status quo” (Figure 1(a), Con). Increases in workload might be tolerated by a balanced enlargement of the heart until the workload exceeds the physiological cardiac capability to respond appropriately. Then cardiac remodeling is initiated and alterations of the cellular and extracellular protein composition turn into a major burden disturbing the structure-function relationship (Figures 4(a) and 4(b)). As a consequence alterations in the protein composition cause changes in hemodynamic load and vice versa. Patients with aortic stenosis show an increase in the thickness of the left ventricular wall, and the heart appears to be enlarged with an overall progression in mass of the ventricle and septum (Figure 1(a), HT). When the load is persistent the heart might undergo an irreversible decompensation and dilation [8, 21]. Myocardial structural changes observed in patients with aortic stenosis can be mimicked in a mouse model of transversal aortic constriction [20, 22]. Within the first three weeks the heart develops compensatory hypertrophy (Figure 1(b), HT), and if the heart is not released from hemodynamic overload, as seen in human patients, the chronic maladaptive response leads to cardiac dilation (Figure 1(a), HT) and potentially HF.

Another major cause of HF is myocardial infarction. Upon chronic underperfusion a complex remodeling process takes place, influencing chamber size, shape, and ventricular function. The ongoing global remodeling leads to an enlargement of the lumen diameter as well as a reduction in the thickness of the left ventricular chamber and septum (Figure 1(a), MI). The human pathophysiology of an acute myocardial infarction can be mimicked in mice by ligation of the left anterior descending artery which interrupts blood flow and causes ischemia in the proximal area. Ventricular performance is lost in the ischemic area and cardiac remodeling leads to increased ventricular volume, chamber dilatation, and thinning of the walls (Figure 1(b), MI). When the infarct size and location are not too severe, the remaining cardiomyocytes might compensate the ventricular wall stress by eccentric hypertrophy.

A further chronic HF condition is termed dilated cardiomyopathy which is characterized by impaired contractility, thinning of ventricular walls, and increases in heart diameter and lumen size, resulting in the dilation of the ventricles (Figure 1(a), DCM). Despite this common phenotype of dilated cardiomyopathy the underlying cause is often linked to viral infections, inherited familial genes, autoimmune disease, or low-level chronic inflammation among others. In addition to cell death cardiomyocyte degeneration, involving the loss of the contractile machinery, has been documented in patients during the transition to HF and is an essential part of cardiac remodeling and dedifferentiation at different stages of DCM (Figure 1(c)) [6, 9, 13, 16, 23]. The increased presence of macrophages combined with elevated levels of various chemokines and cytokines such as MCP-1 (monocyte chemotactic protein-1), TNF-*α*, and oncostatin M during the pathogenesis of dilated cardiomyopathy underscores the contribution of cardiac inflammation [6, 15, 24–26]. As a powerful chemoattractant MCP-1 directs monocytes/macrophages to the sites of injury thereby interfering with regeneration and remodeling of the myocardium [27, 28]. Adverse and harmful invasion of the heart by monocytes/macrophages is mimicked by the cardiomyocyte-restricted overexpression of MCP-1 in genetically modified mice. This strain develops inflammatory dilated cardiomyopathy (Figure 1(b), iDCM) and dies around 6 months due to massive infiltration of the heart and the release of inflammatory cytokines [15, 24]. Similar to human patients, the ventricular cavities of the inflamed heart are enlarged while the thickness of septal and ventricular wall appears to be reduced.

## 3. Identification of Novel Biomarkers and Therapeutic Targets on an “Omics” Platform

Presently the only biomarker in common clinical use for diagnosis and monitoring of HF is B-type natriuretic peptide (BNP), which is released from myocardium undergoing wall stress [29]. Although BNP has prognostic value and no significantly superior alternative HF marker is presently available [29], the usefulness of BNP as a HF marker is limited since it is also released into the circulation under various other disease conditions such as pulmonary embolism and ventricular hypertrophy. A recently described HF marker is fibroblast growth factor-23 (FGF23) [30, 31]. Increased levels of circulating FGF23 were observed in patients with systolic HF, and increased cardiac transcript and protein levels were detected in patients with myocarditis, ischemic cardiomyopathy, and dilated cardiomyopathy [30, 31]. Despite the potential of FGF23 to serve as a HF marker the increase of circulating FGF23 is often associated with faster progression of chronic kidney disease and a higher mortality of hemodialysis patients [32]. Probably the most widely applied biomarker tests are those related to myocardial infarction [29]. However, ischemic markers such as troponins, creatine kinase, and myoglobin are only detectable hours after the ischemic damage and make it impossible to rescue necrotic tissue. Despite their enormous value as diagnostic tools in everyday clinical practice, these assays might not necessarily be helpful, since their specificity and sensitivity are not guaranteed. Therefore, in addition to a comprehensive understanding of a structure-function relationship, there is a need to develop a complementary biomarker toolbox in order to better define the transition from compensatory adaptation to HF via stage-specific biomarker with the further goal of identifying novel drug targets. For this purpose a large-scale screen utilizing “omic” tool is necessary. Ideally, the suffix “omics” addresses the simultaneous analysis of the entire set of biological molecules in a certain field, which is defined by a prefix: proteomic and transcriptomic tools will be featured in this review. Generally there are two aims of “omic” studies: firstly, molecules which are causally involved in heart disease can be targeted by therapeutic intervention and, secondly, molecules which are altered in a predictable manner in response to the disease status can be used as stage-specific markers [29].

**Figure 2:**
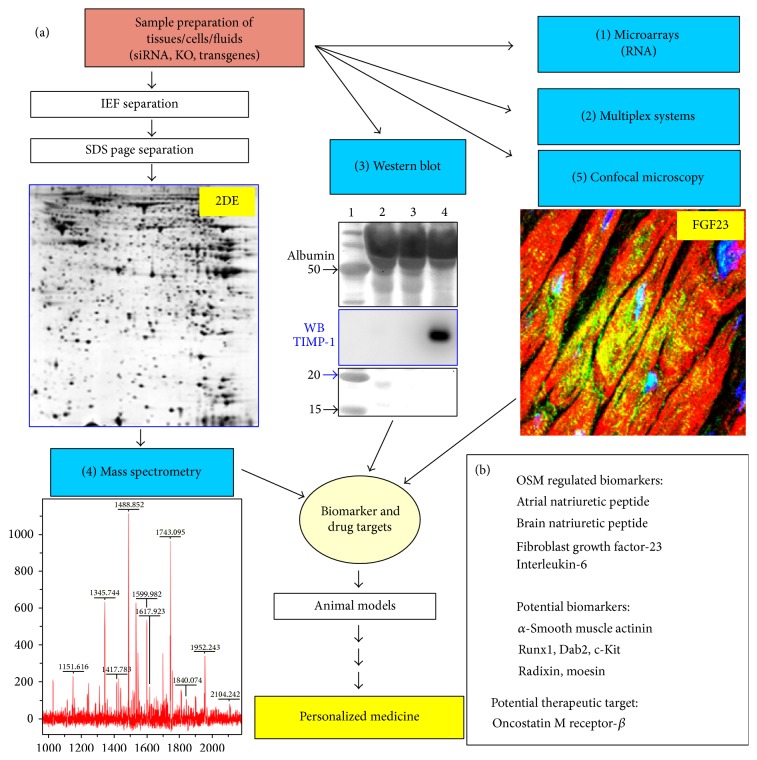
The design of an “omics” technology research platform consisting of complementary core facilities for the future of a personalized medicine (adapted and modified from the “Venia legendi” work of J. Pöling, 2013). (a) Multiplex systems, Western blot, and confocal microscopy are antibody based methods. The Western blot shows a stained membrane resolving 1 *μ*L of two serum samples (2&3) and 1 *μ*L of pericardial fluid (PCF) from a patient with HF and high myocardial level of oncostatin M (size marker was run parallel in 1). A strong signal of TIMP-1 was detected in PCF after antibody hybridization of the membrane and subsequent chemiluminescence detection (WB). When targets are not known the separation of proteins lysates by 2-dimensional gel electrophoresis (2DE) reveals thousands of not yet defined protein spots after silver staining (proteome analysis). Here, a cytoplasmic cardiac 2DE of a 3-day-old rat is shown. Then, gels are scanned and compared and a computer based software program identifies regulated spots. (4) The protein spot is excised and identified by mass spectrometry combined with database searches. (1) In addition RNA might be extracted from the same sample and expression levels of ten thousands of genes can be simultaneously analyzed on DNA microarrays (transcriptome analysis). (5) Confocal microscopy verifies observations and provides further information about the protein. A confocal image shows FGF23-positive cardiomyocytes in a patient with aortic stenosis and high myocardial level of oncostatin M. (b) ANP, BNP, interleukin-6, and FGF-23 were identified as oncostatin M-regulated biomarkers on this platform. Further potential biomarkers such as radixin and moesin are indicated.

An ideal “omics” platform consists of complementary core facilities in order to obtain as much information as possible from fluids, cell cultures, and tissue samples (Figure 2(a)). Fluids are flush-frozen in liquid nitrogen after centrifugation and removal of insoluble material. Usually fluids do not need further processing and can be directly analysed by commercially available ELISA kits (single protein detection by specific antibodies in one sample) or kits using multiplex systems. Multiple cytokine and chemokine assays are multiplex bead-based assays able to simultaneously quantify up to 32 (or even more) targets in one sample. The main advantage of multiplex systems lies in the speed (data are obtained on the same day with little working effort) and sensitivity of determinations (down to picogram levels of proteins). Furthermore, a much lower amount of sample is needed compared to an ELISA due to simultaneous detection of multiple proteins. “Multiplexing” is especially powerful when using highly diluted samples, such as fluids, but might have limitations in the study of tissue biopsies that have to be homogenized in buffers containing detergents. Limitations are further seen in the number of commercially available kits containing panels of different antibodies recognizing predefined protein targets, and the specificity and accuracy of these multiplex systems might be sometimes a matter of concern.

Alternatively protein samples from either tissues or cell cultures can be processed by 2-dimensional gel electrophoresis. After homogenization in an appropriate buffer protein lysates are first separated due to their isoelectric point (isoelectric focusing), which is followed by a separation in the second dimension according to their size (gel electrophoresis). Depending on the complexity of the sample this method is able to separate thousands of protein spots from a single sample in one gel (2DE, Figure 2(a)). Proteins are visualized by staining and gels are scanned and analysed with the corresponding software. Regulated proteins are excised and identified by mass spectrometry (MS). Complementary equipment such as HPLC to enrich certain proteins is often used. The main advantage of the 2DE-based mass spectrometry is the potential to analyse thousands of protein spots simultaneously and to discover newly regulated proteins, in contrast to multiplex detections and Western blot with predefined targets. This allows for large-scale screening of not yet defined biomarkers and drug targets in contrast to the antibody-based assays. Furthermore, the quality of present 2DE technology has improved to the point that once a protein spot has been identified in a gel set there is often no additional need for further MS identification. Limitations are seen in the exclusion of certain protein groups (due to hydrophobicity or size) and in the sensitivity (detection of a protein spot at the nanogram level) when no intermediate step such as HPLC enrichment is performed, and the work is often labour-intensive.

For these reasons, recent developments in proteomics have moved from traditional 2DE methods to gel-free systems [33]. Complex protein mixtures obtained from tissue, cell culture, or plasma samples are digested to peptides and separated by microcapillary reverse phase chromatography before introducing these peptides into the mass spectrometer. After detection of the eluted peptides with the mass spectrometer over a wide m/z (mass-to-charge ratio) range, individual peptides are then selected within the mass analyzer and further fragmented into a ladder of smaller molecules, which result in a second mass spectrum. By repeating this process in automatic routines thousands of MS/MS spectra are obtained, which lead to the identification of thousands of protein species by employing database search programs that match the measured peptides to their corresponding proteins. However, often the complexity of protein mixtures as well as the high abundance of some protein species, such as albumin in serum or actin in cell lysates, hampers the detection capacity of low-abundant proteins. In addition, quantification and comparison of protein expression levels in different samples are often difficult for MS-based gel-free systems.

Transcriptome analysis can also contribute valuable information to the discovery of new biomarkers, being capable of identifying changes in gene expression levels between different study groups, thus pinpointing candidate genes that may be up- or downregulated in a disease situation. DNA microarrays are particularly useful tools in this aspect: after collecting RNA samples from either tissue or cell cultures, cDNA is produced via a retrotranscriptase and hybridized with DNA probes attached to the surface of a manufactured chip. The degree of hybridization is then measured for every DNA probe and, since every gene is represented by multiple specific probes in the chip, an average expression level is calculated for each gene present in the DNA microarray [34].

Although undoubtedly useful, one of the main drawbacks of DNA microarray technology is its dependency on knowledge of the genomic sequence of the species to be studied. For the human genome as well as for some known animal genomes such as rat and mouse commercial DNA microarrays are available while for most animals arrays cannot be obtained. In this case sequence-based technologies that directly determine the nucleic acid sequence are a better alternative, as technological advances have drastically lowered the cost of this type of analysis. RNA-seq consists of the extraction of RNA, its conversion to cDNA, and subsequent fragmentation before sequencing through next-generation sequencing (NGS) technologies and bioinformatic analysis [35]. In any case, candidates identified through either DNA microarray or RNA-seq analysis should be confirmed, usually by first measuring the differential expression of the genes through reverse transcription quantitative real-time PCR (RT-qPCR) and then performing protein analysis.

A further cornerstone in the analysis of proteins is the continuing development of the Western blot technology. The enormous increase in the daily availability of new antibody products (as companies are responding to the challenge to develop antibodies against every single human protein), the increase in sensitivity of detection solutions as well as of imaging systems (Figure 2(a)) to visualize antibody targets (detection at low femtogram level), and the development of semiautomated systems in order to increase throughput provide excellent tools to perform proteome studies at midscale. The specificity and sensitivity of the Western blot approach often allow for a fast and selective analysis of molecular pathways within days, employing small equipment available in almost every laboratory without going through the whole proteome by MS. Once a signalling cascade (e.g. Erk1/2) has been identified this pathway can then be analysed in detail by a set of specific antibodies (Ras, Raf, MEK, etc.). A further significant advantage of Western blot over MS is the fact that the same antibodies can be utilized for confocal microscopy analysis, adding important information on the localization and expression pattern of the analytes in a certain tissue. However, the expense of individual antibodies and lack of a more advanced automation of Western blots significantly reduce the performance of this technique at large scale.

## 4. Primary Cultures of Animal Cells as a Tool to Clarify Disease Mechanisms and Accelerate the Discovery of Novel Biomarkers and Drug Targets

The complex proteome and transcriptome of the heart complicate the extraction of valuable information out of the huge amount of obtained data and simplification is needed. In our experience, the identification of disease-relevant proteins by an “omics” approach in a certain cardiac cell type might be hampered by the abundance of proteins of interest in other cell types. This was the case when we analyzed ezrin-radixin-moesin (ERM), a family of proteins crosslinking actin filaments with the plasma membrane, thus playing a role in cell motility and cell shape maintenance [36]. When ERM protein expression was assessed in samples from patients who have been diagnosed as suffering from dilated cardiomyopathy we found a reduction of moesin by a large-scale Western blot screen [6]. However, confocal microscopy revealed a reexpression and strong accumulation of moesin in cardiomyocytes of the same patients [14]. In order to explain this apparent contradiction we analyzed the expression pattern of moesin in heart tissue by confocal microscopy and found that smooth muscle and endothelial cells of vessels strongly expressed moesin but these vessels were reduced in number in the analyzed patients with DCM. We concluded that the reduced amount of moesin in the human diseased myocardium was due to the depletion of vessels, which masked the reexpression of moesin in cardiomyocytes [14].

**Figure 3:**
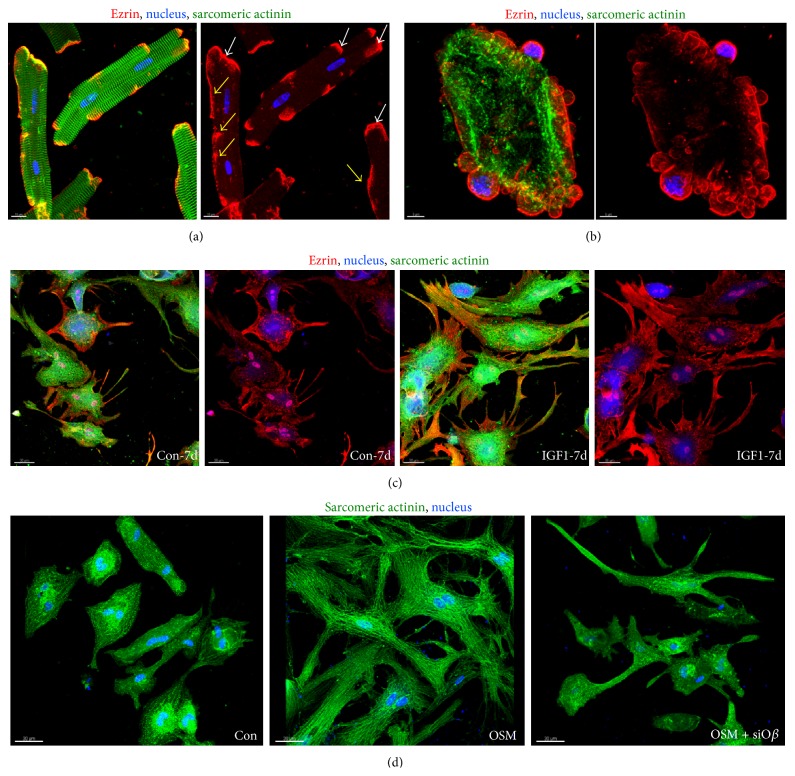
Cardiomyocytes respond to stress by membranous translocation of ERM proteins. (a) Fluorescence micrographs of freshly isolated adult rat cardiomyocytes (4 hours) show different degrees of ezrin translocation (yellow arrows). Ezrin is usually located at the intercalated disc (white arrows) but upon translocation it is detected laterally of the membrane. (b) Fluorescence micrographs demonstrate massive translocation of ezrin. Ezrin is part of the cell blebs which are, when occurring to this extent, characteristic for dying cells. (c) Fluorescence micrographs show increases and accumulation of ezrin in cell extensions of IGF-1 stimulated adult cardiomyocytes after seven days. Note that serum shows also some effects on ezrin localization in control cultures (Con). (d) Fluorescence images of oncostatin M receptor-*β* siRNA treated adult rat cardiomyocytes (OSM + siO*β*) in culture demonstrate successful interruption of OSM induced remodeling after 7 days. Con indicates albumin treated control cultures.

**Figure 4:**
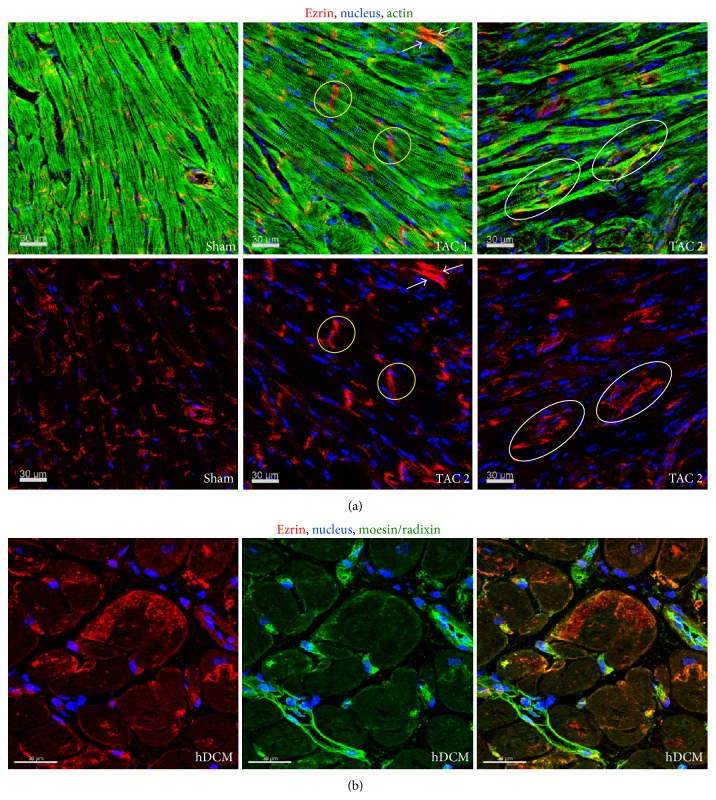
Spatial distribution of ezrin during adaptation and HF. (a) Longitudinal sections of the mouse myocardium 1 month after transaortic constriction (TAC). In sham operated animals ezrin shows a regular appearance at the intercalated disc, which is disturbed to a variable degree in mice after TAC (yellow circles). In the fluorescence micrographs cardiomyocytes show different degrees of ezrin translocation in cardiomyocytes (white arrows (TAC1) and oval circles (TAC2)). These animals recover after the release of constriction. (b) Fluorescence micrographs demonstrate massive lateral accumulation of ezrin in patients with end-stage HF. This pattern of moesin and radixin labeling corresponds with that previously described [14].

In order to accelerate the discovery of disease-relevant peptides animal culture models provide “simplified” systems for the analysis on an “omics” platform (Figure 3(a)). Primary cultures of cardiomyocytes are particularly suitable for “omics” studies, since adult cardiomyocytes are terminally differentiated and they transfer their epigenetic, genomic, and proteomic* in vivo* status into the culture dish, which might not be preserved in cell lines or passaged cells [37]. Primary cultures are, as defined by the Latin term “primus,” cells which are directly used for experiments after isolation from the animal and not further passaged. The strong correlation we demonstrated between differentially regulated proteins of remodeling cardiomyocytes* in vivo* and* in vitro* [6, 14, 15] has permitted us to find ERM proteins (Figures 3 and 4) and other potentially relevant candidates [6, 15, 31] in oncostatin M (Figure 2(b)) and IGF-1 (insulin-like growth factor-1) stimulated cultures of adult rat cardiomyocytes.

Despite their strength as “pumping units” adult cardiomyocytes are rather fragile and a good quality of cells can only be obtained when the heart is perfused on a Langendorff apparatus and collagenase disrupts the extracellular matrix, thus releasing rod-shaped cardiomyocytes [38]. The susceptibility of these cells to mechanical stress reflects the need of the cardiomyocyte to adapt to changes in cardiac architecture in order to avoid harmful mechanical distortion. In culture, remodeling of cardiomyocytes already starts within the first hours (Figures 3(a) and 3(b)) when ERM proteins accumulate at membrane areas different from the intercalated disc. When stimulated for one week with IGF-1, cardiomyocytes increase significantly in size and ezrin is mainly localized at the cellular extensions (Figure 3(c)) probably stabilizing tension during the growth process. In addition, cytoplasm and the nuclei were positive for ezrin. When the mechanical stress suffered during the isolation process becomes too severe freshly isolated cardiomyocytes lose their highly organized three-dimensional structure, round up, and die by blebbing (Figure 3(a) versus Figure 3(b)). Ezrin-positive blebs can be easily recognized as spherical protrusions of the membrane (Figure 3(b)). We made similar observations with moesin and radixin after stimulation with oncostatin M indicating that the translocation of ERM proteins belongs to a “rapid adaptive stress program” to compensate unfavorable mechanical distortion [14].

In order to understand whether our observations are also true in the remodeling myocardium we took advantage of the TAC hypertrophy model (Figure 4(a)). In the normal myocardium ezrin localizes to the intercalated disc. After 1 month of transaortic constriction—a time point when the heart is still able to recover if the constriction is removed—cardiomyocytes of some myocardial areas are increased in size and depict a diffuse pattern of ezrin in the intercalated disc. Some cardiomyocytes show an unusual localization of ezrin and the pattern resembles the extensions of IGF-1-treated cardiomyocyte cultures (white arrows). In other areas of the myocardium ezrin appears laterally in cardiomyocytes and the intercalated discs are hardly recognizable, indicating that most ezrin is translocated. We conclude that ezrin translocation may serve as a mechanism to stabilize cardiomyocytes in the pressure-overloaded myocardium. In the human failing heart ERM proteins show an unusual localization and expression pattern. In contrast to the healthy heart, moesin is reexpressed in cardiomyocytes [14] and ezrin shows massive membranous and cytoplasmic accumulation. Taken together, these are signs that the cardiac damage led to a structural remodelling of cardiomyocytes, although this adaptive attempt did not prevent HF.

A second advantage of animal cell culture systems resides in the possibility of selecting from the multitude of cardioactive substances individual cytokines and performing functional studies. By these means we have identified oncostatin M as a major modulator of cardiac remodeling, as well as a promising therapeutic target in dilated cardiomyopathy. We discovered that oncostatin M promotes* in vivo* functional deterioration and lethality when chronically activated [6, 15]. In addition pharmaceutical or genetic targeting of the oncostatin M receptor-*β* (O*β*) attenuated HF and reduced mortality in mice model with dilated cardiomyopathy [6, 15]. Furthermore, the relative ease in performing specific knock-downs of proteins by siRNA in adult cardiomyocytes can in certain aspects be an alternative to the cumbersome construction of knock-out mice. As an example, Figure 3(d) shows how a simple knock-down of the oncostatin M receptor-*β* (O*β*) by siRNA blocks the remodelling effect of oncostatin M on adult cardiomyocytes. Taking advantage of this fact, we identified by an siRNA-based knock-down of various signalling cascades, combined with an extensive Western blot analysis, that remodeling of oncostatin M-stimulated adult cardiomyocytes depends on Erk1/2 but not on p38 or SAP kinase pathways [6]. In the same study we observed markedly increased amounts of O*β* in patients with dilated cardiomyopathy. However, this cytokine is hardly detectable in the circulation and is, therefore, unsuitable to serve as a circulating biomarker. Since oncostatin M itself induces the expression of a variety of peptides in cultured cardiomyocytes, a transcriptome and proteome analysis of OSM-stimulated cardiomyocytes was performed for novel disease-relevant molecules. Among more than 500 strongly regulated proteins we identified radixin, moesin, ANP, BNP, and the upcoming HF marker FGF23 [6, 14, 15, 31] (Figure 2(b)). This observation was insofar surprising since it was assumed that the bone, but not the heart, is the main source of circulating FGF23 in heart diseases. The discovery of FGF23 on the transcript as well as protein level in the failing heart underlines the power of an “omics”-based initial analysis of primary animal cell cultures [31].

## 5. Conclusions

Despite the enormous efforts put worldwide into HF research, the number and specificity of cardiovascular biomarkers and targets are still regarded as dissatisfactory [3, 10, 29, 39]. However, the combination of well-established traditional methodologies with sophisticated “omics” toolboxes generates a highly promising platform and inspires great hope for the medicine of the future. High-throughput technologies are ideally suited to identify genes and proteins involved in heart diseases that may have gone undiscovered so far. Gene expression screens, such as DNA microarrays or RNA-seq, as well as large-scale protein expression profiling through 2DE or gel-free systems combined with mass spectrometry, generate an enormous amount of data from which novel therapeutic strategies and potential targets can be selected through careful bioinformatic analysis. Although undoubtedly powerful, large-scale screens still require the validation and further study of individual candidates through more traditional methods, such as Western blot and electron and confocal microscopy, in order to verify, localize, and characterize novel biomarkers and drug targets. Moreover, a mere “omics” approach of human tissue samples frequently leads into a “data jungle” hiding valuable disease-relevant clues. For these reasons, the selection of specific animal models such as transversal aortic constriction as a model for patients with aortic stenosis reduces the complexity of the human pathology and allows for a more focused analysis. The analytical capacity of these animal models might be expanded by genetically modified mice such as transgenes or knock-outs, which permit the understanding of disease-relevant processes. The greatest reductions in complexity offered by primary animal cell cultures might make them an ideal start-up on an “omics” platform before moving to* in vivo* models.
